# Body Fluid Identification in Samples Collected after Intimate and Social Contact: A Comparison of Two mRNA Profiling Methods and the Additional Information Gained by cSNP Genotypes

**DOI:** 10.3390/genes14030636

**Published:** 2023-03-03

**Authors:** Helen Johannessen, Erin Hanson, Peter Gill, Cordula Haas, Erik Francisco Bergseth, Jack Ballantyne, Ane Elida Fonneløp

**Affiliations:** 1 Department of Forensic Medicine, University of Oslo, 0315 Oslo, Norway; 2 National Center for Forensic Science, University of Central Florida, Orlando, FL 32826, USA; 3 Department of Chemistry, University of Central Florida, Orlando, FL 32816, USA; 4 Department of Forensic Sciences, Oslo University Hospital, 0372 Oslo, Norway; 5 Zurich Institute of Forensic Medicine, University of Zurich, 8057 Zurich, Switzerland; 6 Centre for Ecological and Evolutionary Synthesis (CEES), Department of Biosciences, University of Oslo, 0371 Oslo, Norway

**Keywords:** sexual assault cases, transfer, persistence, mRNA, body fluid identification, cSNPs

## Abstract

The ability to associate a contributor with a specific body fluid in a crime stain can aid casework investigation. The detection of body fluids combined with DNA analyses may supply essential information, but as the two tests are independent, they may not be associated. Recently, the analysis of coding region SNPs (cSNPs) within the RNA transcript has been proven to be a promising method to face this challenge. In this study, we performed targeted RNA sequencing of 158 samples (boxershorts, fingernail swabs and penile swabs) collected from 12 couples at different time points post-intimate contact and after non-intimate contact, using the Ion S5™ System and BFID-cSNP-6F assay. The aim of the study was to compare the performance of the MPS and CE methods in the detection of mRNA markers, and to associate body fluids with contributors by their cSNP genotypes. The results of the study show a lower success rate in the detection of vaginal mucosa by the MPS compared to the CE method. However, the additional information obtained with the cSNP genotypes could successfully associate body fluids with contributors in most cases.

## 1. Introduction

The detection of body fluids, combined with DNA analyses, can supply essential information about a crime stain. If a crime stain provides a positive test result for saliva and a DNA profile that matches a suspect’s reference profile, the findings support the proposition that the saliva and DNA originate from the suspect. The tests for body fluid detection and DNA profiling are performed separately, based on specific proteins in body fluids [1,2] and short tandem repeats (STRs), respectively, in the DNA molecule [3] (pp. 85–90). The two tests provide independent information, and the results may not be associated with each other. Consequently, a donor may not necessarily be attributable to a body fluid detected in a mixed DNA sample. This may cause challenges in the investigation, especially if the body fluids are not gender-specific, or if the DNA sample originates from multiple contributors of the same gender.

mRNA analysis can detect several body fluids simultaneously [4,5,6,7], and this has been successfully demonstrated by several methods, particularly by Endpoint PCR/Capillary Electrophoresis (CE) [5,6,7] and Massively Parallel Sequencing (MPS) [8,9,10]. The advantage of MPS is that, in addition to detecting mRNA markers, the nucleotide sequence of the RNA transcript is also achieved. The detection of coding region SNPs (cSNPs) within the mRNA transcript has been introduced as a promising approach to associate a body fluid with a donor [11,12,13,14,15,16,17,18,19]. Comparison of cSNP genotypes within a body fluid-specific transcript to the corresponding genomic cSNP genotypes in reference samples from donors, may be used to associate a donor with a given body fluid. However, previous studies have suggested that more markers for some body fluids, including vaginal mucosa, are needed to improve the discriminatory power of cSNPs [12,17]. In a recent study by Hanson et al. [20], a dual-functional mRNA sequencing panel (“BFID-cSNP-6F assay”) was demonstrated, including 23 mRNA biomarkers covering six body fluids/cell types (blood, semen, saliva, vaginal mucosa, menstrual blood and skin) and 46 cSNPs. In this assay, MUC22 was introduced as a new and promising biomarker for vaginal mucosa, covering seven cSNPs in the BFID-cSNP-6F assay.

In a previous study, we investigated the transfer and persistence of DNA quantity and mRNA vaginal mucosa markers in 158 samples collected from boxershorts, fingernail swabs and penile swabs by 12 couples after intimate contact, and when no intimate contact had occurred [21]. The samples were analyzed using the CE method. In this study, we analyzed the same samples by targeted RNA sequencing with the MPS method using the Ion S5™ System (Ion Torrent^TM^ by Thermo Fisher Scientific, Waltham, MA, USA) and the BFID-cSNP-6F assay [20]. The overall aims of the study were: (1) to compare the performance of the detection of mRNA markers with the CE and MPS methods; and (2) to associate body fluids with contributors by the detection of cSNPs.

## 2. Materials and Methods

### 2.1. Ethical Declaration

The Data Protection Officer (DPO) at Oslo University Hospital approved this study prior to initiating this project (reference 20/13115). The project was carried out according to the approved procedures and protocols, and all participants gave their informed consent.

### 2.2. RNA Extracts and Reference Samples

In this study, 158 RNA extracts and 24 DNA reference samples presented in the paper by Johannessen et al. [21] were analyzed. The sample set consisted of 122 samples (fingernail swabs, penile swabs and boxershorts) collected from 12 couples at 0 (immediately after intimate contact), 12, 18, 24 and 36 h post-intimate contact, and 36 samples collected from the same locations, but with no prior intimate contact. The boxershorts were collected after being worn by male participants for 5–6 h after intimate contact and for the same period with no prior intimate contact. The samples were stored for about 1 year in −20 °C (DNA extracts) and −80 °C (RNA extracts) freezers prior to analysis.

### 2.3. cDNA Synthesis

Complementary DNA (cDNA) synthesis of the RNA extracts was performed using SuperScript^TM^ IV VILO^TM^ Master Mix (invitrogen by Thermo Fisher Scientific, Waltham, MA, USA), following the manufacturer’s protocol. Twenty-five ng (max. 12 µL) of total RNA was mixed with 3 µL Master Mix, resulting in 15 µL reverse transcription reaction volume. Negative reverse transcription controls were not performed, as this was carried out in the former study, and no genomic DNA was detected in any of the RNA extracts [21].

### 2.4. Library Preparation

Automated library preparation was carried out using the Precision ID DL8 kit (Applied Biosystems^TM^ by Thermo Fisher Scientific, Waltham, MA, USA) on an Ion Chef™ Instrument (Ion Torrent^TM^ by Thermo Fisher Scientific, Waltham, MA, USA) according to the manufacturer’s protocol. The RNA (cDNA) samples and DNA samples were prepared separately with the respective 2X primer pools (one primer pool per run) from the BFID-cSNP-6F assay, as described by Hanson et al. [20]. The assay comprised a panel of 23 body fluid-specific mRNA markers for blood, semen, saliva, vaginal mucosa, menstrual blood and skin, as well as 46 cSNPs (Supplementary File S1, Table S1). The primers in the BFID-cSNPs-6F assay are designed not to amplify non-mRNA targets. They span different exon–exon junctions or exons within the amplicon so that the corresponding DNA product will be too large to be generated (>200 bp). The PRM1 semen-specific marker, due to a short intron sequence in the DNA product, result in a genomic DNA (gDNA) amplicon (191 nts) if present. The corresponding RNA/cDNA amplicon is 100 nts. The cDNA reaction product (15 µL) and 2 ng of DNA (in 15 µL volume) were added to the IonCode^TM^ 96-well PCR plate. The AmpliSeq^TM^ workflow options were set to 1 primer pool, 24 target amplification cycles and 4 min annealing and extension time.

### 2.5. Library Quantification

The combined libraries were quantified with the Ion Library TaqMan^TM^ Quantification kit (Ion Torrent^TM^ by Thermo Fisher Scientific, Waltham, MA, USA) and the 7500 Real-Time PCR System (Applied Biosystems^TM^ by Thermo Fisher Scientific, Waltham, MA, USA), according to the manufacturer’s protocol. Three dilutions (1:1, 1:20 and 1:100) of each combined library were analyzed, and the concentration was calculated from the least diluted sample that fell within the standard curve.

### 2.6. Template Preparation and Sequencing

Template preparation and sequencing were carried out using an Ion S5^TM^ Precision ID Chef & Sequencing Kit (Applied Biosystems^TM^ by Thermo Fisher Scientific, Waltham, MA, USA) on Ion Chef™ Instrument and Ion S5™ System (Ion Torrent^TM^ by Thermo Fisher Scientific, Waltham, MA, USA) according to the manufacturer’s protocol. Combined libraries were diluted to approximately 50 pM, and 4 diluted libraries (comprising barcodes 1–32) were super-pooled by adding equal amounts of each. The super-pooled, combined library was loaded to an Ion 530™ Chip (Ion Torrent^TM^ by Thermo Fisher Scientific, Waltham, MA, USA) and sequenced with 500 flows (200-base read-run) and the Ion’s default samba flow order. The DNA reference libraries were run on a separate chip. Hg19 was used as a reference genome for the DNA reference samples. Custom reference genome for RNA samples, target files (target regions) and hotspot files (variant calling), described in Hanson et al. [20], were applied to the RNA and DNA samples.

### 2.7. Data Analysis

The BAM and BAI files were analyzed with the Ion Torrent Mapping Alignment Program (TMAP) [20]. The program requires a nucleotide from each of the exons in the amplicon (almost a full read length), and subsequently maps them to the mRNA targets. The output spreadsheet includes the total coverage, percentage of body fluid contribution, individual read counts and cSNP genotypes for each sample. A minimum coverage of 100 reads per profile was required, and a threshold of 0.5% of the total reads for each individual read count was applied to reduce noise (set to zero). To designate a body fluid, reads for at least two body fluid-specific mRNA markers were required. cSNP genotypes detected in the RNA sequencing data (RNA-cSNP) were compared to the corresponding genomic cSNP genotypes in the DNA reference samples (DNA-cSNP) from both the donor and the person of interest (female partner). In order to link a body fluid to a participant, all detected cSNPs aligned with the reference sample had to be observed. One allele drop-out of the detected genotypes for a body fluid was accepted in order to allow a match designation. The body fluid was called “inconclusive” if more than one allele drop-out was observed, or if the donor and the person of interest (POI) shared genotypes for the detected cSNPs. If the two participants shared alleles due to one reference showing homozygous genotypes, the body fluid was attributed to the reference with unique alleles that aligned to the RNA-cSNP.

In addition, the mRNA results were compared to those obtained from the sample set run with the CE method, as described by Johannessen et al. [21]. Ten RNA extracts which had previously given a positive test result for vaginal mucosa with the CE method, but a negative result with the MPS method, were re-analyzed with the CE method in order to demonstrate that the storage of the extracts at −80 °C had not significantly reduced the quality of the RNA prior to MPS analysis. Vaginal mucosa markers were detected in all ten samples, and the positive and negative controls gave the expected results.

*t*-tests were carried out to determine whether the expected values were the same between groups at a 5% significance level. Furthermore, linear regression was used to test the association between the RNA quantity and the coverage (number of total reads or reads of the vaginal mucosa markers) of the MPS positive samples. Statistical analyses and figures were made in *R* version 4.1.3 (www.r-project.org) using the *tidyverse* package, version 1.3.1.

## 3. Results

### 3.1. RNA Sequencing and Bodyfluid Identification

**Figure 1:**
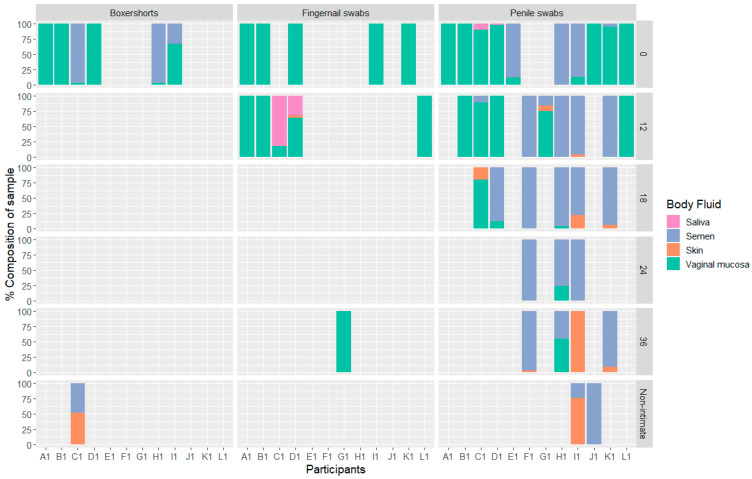
Bar chart showing the percentage of body fluids detected in each sample, divided into participants, sample location and time of sampling, *n* = 52.

Overall, 33% (52) of all analyzed samples gave positive results with the MPS technology and the BFID-cSNP-6F assay (Figure 1). In addition, for 26 samples, reads were obtained, but did not pass the quality thresholds described in Section 2.7. A positive test for vaginal mucosa was observed in 36 intimate contact samples, with the highest success rate in the samples collected at 0 and 12 h. Only six samples tested positive for vaginal mucosa beyond 12 h post-intimate contact. Semen was the next most common body fluid, detected in 27 samples, followed by skin and saliva, detected in 11 and 4 samples, respectively. Of the samples collected after non-intimate contact, only three showed a positive result where skin and/or semen were detected. Blood and menstrual blood were not observed in any of the samples.

The total number of reads per sample varied from 113 to 419, 848 reads, with a median of 3360 reads. The distribution of reads per body fluid for each sample is presented in Supplementary File S1, Figure S1.

**Figure 2:**
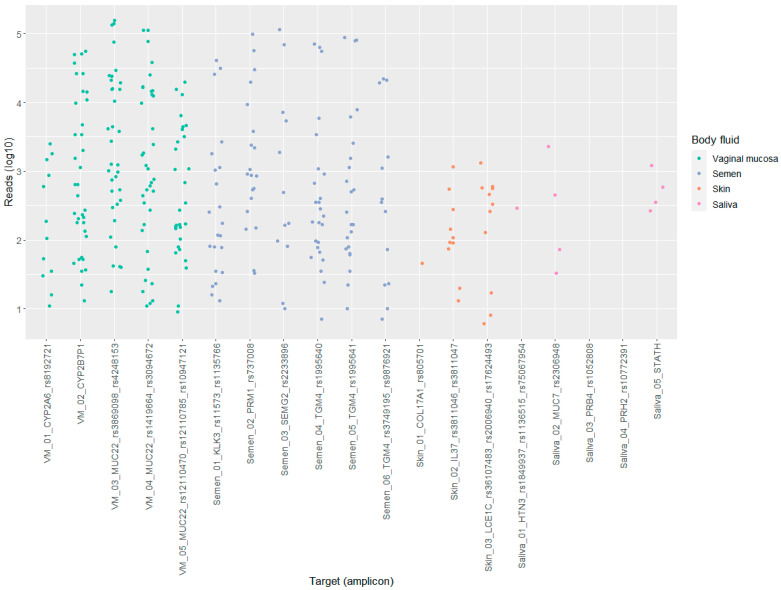
Scatter plot displaying the distribution of reads (log10) per targets (amplicon) for saliva, semen, skin and vaginal mucosa (VM), *n* = 36.

The number of reads per target (amplicon) in the BFID-cSNP-6F assay show a comparable distribution between the semen and vaginal mucosa targets (Figure 2). In comparison, the saliva and skin targets produced lower reads.

There was a significant association between the RNA quantity detected in the samples and the total coverage or the total number of reads for the vaginal mucosa markers (linear model, *p*-value = 0.026 and 0.0018, respectively). However, only 9 and 25% of the data, respectively, could be explained by the coefficient of determination (R^2^-value); see Supplementary File S1, Figures S2 and S3. Only about half of the RNA extracts reached the optimal 25 ng input for the MPS method (Supplementary File S3).

**Figure 3:**
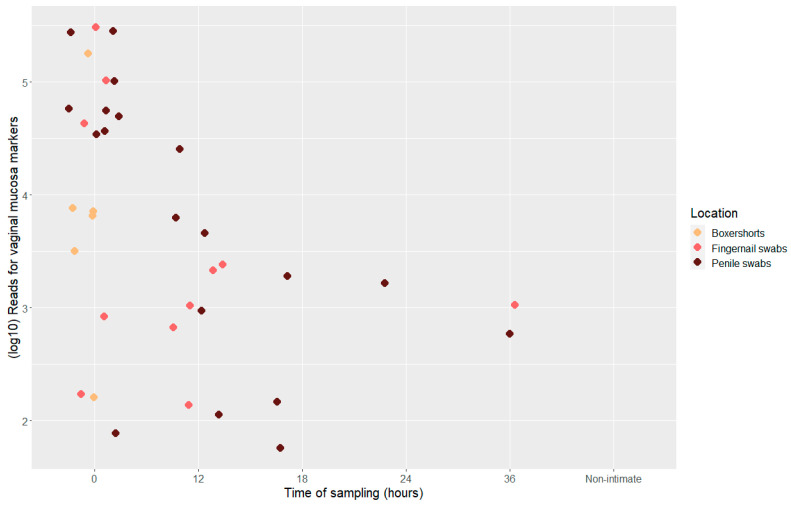
Scatter plot displaying total number of reads (log10) for vaginal mucosa markers, divided into location and time point of sample collection, *n* = 36.

The number of total reads for the vaginal mucosa markers varied from 57 to 305, 813 reads (Figure 3). The number of reads decreased significantly between samples collected at 0 and 12 h post-intimate contact (*t*-test, *p* = 0.0136); the data from beyond 12 h were too few to perform similar comparisons. CYP2A6 had, overall, the lowest expression in the samples of the three mRNA vaginal mucosa markers (Supplementary File S1, Figure S4).

The gDNA PRM1 marker was detected in 7 of the 158 samples (Supplementary File S1, Table S2). In all cases, the gDNA reads were ≤ 1% of the RNA PRM1-specific reads and the total coverage of the profile.

#### Comparison of CE and MPS Technology

All samples were previously analyzed with a 19-plex mRNA body fluid panel using the CE method [21]. The results of the mRNA body fluid identification by both methods (CE and MPS) are summarized in Table 1. The success rate of the detection of vaginal mucosa decreased by almost 50% with MPS compared to CE. Of the 68 samples detected to be positive for vaginal mucosa with CE, 32 were detected positive with MPS. Of the remaining CE-positive samples, MPS found 4 samples to be positive for semen only and 1 sample to be positive for semen and skin, but 31 samples were negative for all body fluids. On the other hand, four of the samples that tested positive with MPS were not positive with CE, but were sporadically detected (i.e., <50% of vaginal mucosa markers observed). All non-intimate contact samples gave negative results for vaginal mucosa with MPS, while a pair of boxershorts collected after non-intimate contact showed a positive result with CE. The detection rates for semen were more comparable, with 33 and 27 positive samples for CE and MPS, respectively. Twenty samples were positive with both methods.

### 3.2. Coding Region SNPs Identification

Overall, 46 cSNPs are located within 20 of the 23 genes in the BFID-cSNP-6F assay. The results of the comparison of the cSNPs in the samples and the reference samples from the participants are summarized in Table 2, where “POI” refers to the female partner and “donor” to the male partner. In 97 and 85% of the positive samples for vaginal mucosa and semen, respectively, the cSNP genotype information could associate the body fluid to the POI (vaginal mucosa) or to the donor (semen). Only a few samples were called “inconclusive” for vaginal mucosa and semen, and neither of the two body fluids were associated with the “wrong” participant. Of the positive samples for saliva and skin, POI could only be attributed to skin in one sample (penile swab collected 12 h post-intimate contact). In the remaining samples, saliva and skin were either associated with the donor or called “inconclusive” (Table 2). Alleles that could not be explained by either the donor or POI were not observed in any of the samples.

Two of the mRNA vaginal mucosa markers, CYP2A6 and MUC22, were associated with one and seven cSNPs, respectively, within the BFID-cSNP-6F assay. The CYP2B7P1 marker does not contain any cSNP. The number of reads for the eight vaginal mucosa cSNPs were in the same range, from approx. 10–100,000 reads (Supplementary File S1, Figure S5).

Two examples of cSNP results are given in Table 3. The first example is a penile swab collected 24 h post-intimate contact. In this sample, several of the body fluid-specific genotypes only corresponded to one of the participants in the couple, allowing easy association of the body fluid with the contributor. In the second example, a penile swab collected immediately after intimate contact, the genotypes of the vaginal mucosa markers were associated with the POI, while the saliva was called “inconclusive” as both the donor and the POI shared the same genotype for the observed cSNP.

In cases where allele drop-out was observed (the sample genotype was observed as homozygous, but the reference sample from participant was heterozygous), most of the reads for these cSNPs were low, typically below 100 reads for the detected allele, but ranged within 7–689 reads.

Allele drop-in was observed for two samples. In one 0 h post-intimate-contact penile swab, in addition to the genotypes that aligned with the POI for vaginal mucosa markers, an allele of 143 reads was detected for one of the cSNPs (rs10947121) in the MUC22 marker. In comparison, the alleles aligned with the POI for this mRNA marker had 3905 reads or higher. Likewise, an additional allele of 31 reads was observed for one of the cSNPs (rs36107483) in the LCE1C skin marker in a boxershorts sample collected after non-intimate contact. The other alleles, which aligned with the donor, were in the range of 33–97 reads for the detected skin markers. In both of these cases, the drop-in allele could be observed in the partner’s reference sample.

### 3.3. The Association of mRNA and DNA Quantity of POI

**Figure 4:**
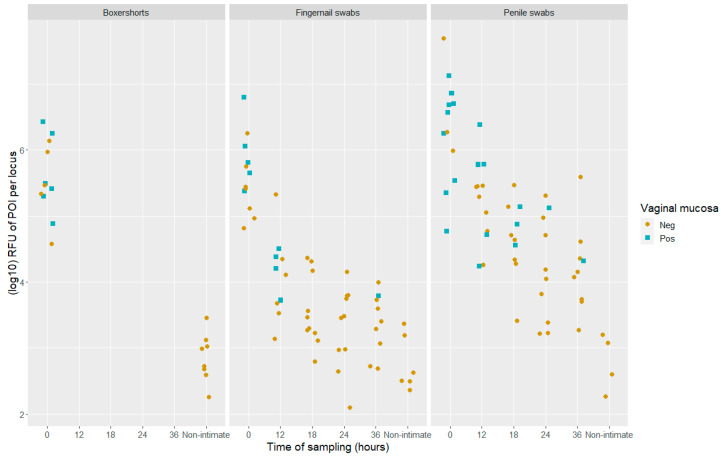
Scatter plots of DNA quantity ($\log_{10}{\bar{RFU}}_{POI}$) in positive samples (LR > 10,000) which gave a positive (blue) or negative (light brown) test result for vaginal mucosa with the BFID-cSNP-6F assay and MPS technology. The samples are divided into locations and time of sampling, both post-intimate contact and non-intimate contact, *n* = 133.

The DNA quantity, measured in adjusted RFU value aligned with POI per locus ($\log_{10}{\bar{RFU}}_{POI})$, for all samples (DNA fractions) were presented by Johannessen et al. [21]. In their study, a sub-source LR threshold of 10,000 was applied to describe a positive finding corresponding to DNA results that supported the proposition that the POI was a donor. The combined values of DNA quantity and the test results for vaginal mucosa are presented in Figure 4. The DNA quantity was significantly higher in samples with a positive test result for vaginal mucosa compared to the samples that gave a negative test result (*t*-test, *p* < 0.0004). However, the association between a positive test result and the DNA quantity of the POI is less distinct compared to the previous study that used the CE method (Figure 6 in ref. [21]).

In 35 of the 36 samples that tested positive for vaginal mucosa, the POI was detected in the DNA fraction. A single sample, a 12 h post-intimate-contact fingernail swab, gave a positive test result for vaginal mucosa, but the sub-source LR value was 728. Thus, this was classified as a negative sample in accordance with the applied threshold of 10,000. The genotype information gained from the detected cSNPs was “inconclusive” for vaginal mucosa when compared to the reference samples.

## 4. Discussion

In this study, we performed RNA sequencing of 158 samples from fingernail swabs, penile swabs and boxershorts collected after intimate and non-intimate contact using the Ion S5™ System and the BFID-cSNP-6F assay. The study was carried out in order to detect body fluids present in the samples and to associate the body fluid to the contributor (donor or female partner). The assay has previously performed successfully on neat body fluids and cell types [20], and in this study, we have shown that the assay can also perform well on “mock case” samples of the kind that are likely to be submitted for analysis in casework.

Of the 158 samples, 52 provided results with the BFID-cSNP-6F assay according to our scoring rules. The threshold to categorize a sample as positive was set to a minimum coverage of 100 reads per profile, individual counts of a minimum of 0.5% of the total reads and reads for at least 2 mRNA body fluid-specific markers. If considering all reads, and only applying the 0.5% threshold of total reads, 26 additional samples would have been reported. However, many of these samples produced low coverage and reads in only one mRNA marker. The latter included observations of a vaginal mucosa marker in several of the non-intimate contact samples, or the cSNPs information inferring the male donor to be the contributor for the only detected vaginal mucosa marker (unspecific expression). In addition, heterozygous imbalance (*H_b_* < 0.5) in the cSNP genotypes was often observed; in some markers, an extreme *H_b_ <* 0.1 was recorded, mainly in the TGM4 semen marker and the MUC22 vaginal mucosa marker (Supplementary File S1, Figure S6). In the study by Hanson et al. [20], in which an individual count threshold of 500 reads was applied for this assay, heterozygous imbalance of these markers was also reported. Due to the uncertainties observed in the data, we chose the additional thresholds of minimum 100 reads per profile and minimum 2 body fluid-specific markers in order to improve our confidence in reporting true positive samples. Consequently, we lowered the threshold of coverage to compensate for the low level of RNA present in the samples compared to the thresholds applied by Hanson et al. [20].

In this study, 36 samples gave a positive result for vaginal mucosa. The success rate for vaginal mucosa with the MPS method was reduced by almost 50% compared to the CE method [21]. On the contrary, none of the non-intimate contact samples gave a positive result with MPS, although one sample in this category produced a positive test result with CE. Two additional intimate contact samples (0 and 12 h penile swabs) had reads detected for the vaginal mucosa markers, but were classified as negative as they fell below the 0.5% threshold of total reads in the profile (due to the high number of reads in semen markers). For these two samples, both vaginal mucosa and semen were successfully detected with the CE method. The detection rates for semen and saliva were slightly lower with MPS compared to CE. No samples showed positive results for blood or menstrual blood with the MPS method. In comparison, with the CE method, a few samples gave positive result for blood, but none for menstrual blood. With the CE method, all samples were run with three different amounts of cDNA. With the MPS method, samples were run only once, mainly due to the cost of the analysis. The triplicate run included in the CE method may increase the success rate, as there is a better chance of reaching the optimum amount of cDNA for the PCR reaction. The observed differences in analytical sensitivity (i.e., limit of detection) between the MPS and CE platforms were notable, but not entirely unexpected. Although the precise reason for this has not been identified, several possibilities can be envisioned. For example, there appears to be less tolerance for common PCR inhibitors present in challenging samples with MPS compared to CE [22], and, of the three vaginal mucosa markers used in both CE and MPS, two differed between the platforms. Different markers can have different expression levels (and associated variances) in the target fluid. Furthermore, MPS library preparation methods for MPS have not yet been fully optimized for low-level samples.

Although all 158 samples were collected from skin or from fabric that was in close contact with skin, only 7% (11) of the samples gave a positive result for skin. This low rate was in accordance with the findings of previous studies [12,20] which showed that low RNA yields were often recovered from skin samples.

The cSNP genotypes in the BFID-cSNP-6F assay performed well enough to discriminate the contributor of the body fluid. In this study, the positive results were mainly for vaginal mucosa and semen, which are both gender-specific biomarkers. However, as the mRNA markers for body fluid identification are not absolutely specific, meaning that they may be expressed in lower abundance in non-target body fluids, the additional cSNP genotype information provides additional support for the inferred body fluid type. This would also be the case if a suspect had sexual intercourse with another female prior to an alleged rape case, and the additional information of the vaginal mucosa cSNPs (e.g., from a penile swab) could help to identify the person from whom the detected vaginal mucosa came from. The eight cSNPs within the transcripts of the vaginal mucosa markers CYP2A6 and MUC22 could successfully associate the POI to vaginal mucosa in 35 of the 36 cases. In the last sample, the conclusion was “inconclusive”, since the results inferred several allele drop-outs (all homozygous genotypes with 18–65 reads). The DNA result for the sample gave a LR of 728 in support of the proposition that the POI was a contributor, which was below our reporting threshold of 10,000. The mRNA marker CYP2A6 was, overall, less sensitive compared to CYP2B7P1 and MUC22, suggesting that CYP2A6 is expressed in lower abundance in vaginal mucosa. In two samples, an allele drop-in was observed in one cSNP. One of the latter samples was a penile swab collected immediately after intimate contact, and an additional allele of less than 4% of the reads of the alleles aligned with the POI, was observed for one vaginal mucosa cSNP. The DNA fraction for this sample aligned with the reference samples of the donor and the POI. The other sample was from a non-intimate contact boxershorts sample, and an additional allele was observed for a cSNP in the LCE1C skin marker. This allele had a low read count, but was within the same range as the alleles aligned with the donor. The DNA fraction for this sample showed a single donor profile. For both samples, the additional allele was the only hint inferring a mixture of two contributors of the respective body fluid/cell type; and, hence, was interpreted as a drop-in allele. In 18 samples, alleles of unknown origin were observed in the DNA fraction [21]. None of these samples gained additional information from the cSNP genotypes to help assign which body fluid or cell type the unknown person may have derived from.

The BFID-cSNP-6F assay has the potential to detect microhaplotypes for some of the mRNA markers, e.g., rs3869098 and rs4248153, that are located on the same amplicon as mRNA marker MUC22 [20]. The detection of these microhaplotypes can improve the discrimination power. However, due to the very few observations of phased differences in the microhaplotypes, i.e., genotype AG for both SNPs resulting in haplotype AG/GA (versus AA/GG), in the previous study by Hanson et al. [20], in this study, we chose to focus only on the individual cSNPs.

The RNA sequencing of the sample set resulted in 67% (106 samples) yielding negative results, where 80 samples produced no reads and 26 samples had reads, but did not fulfill the scoring thresholds. A higher success rate was expected of the analysis, especially considering that the BFID-cSNP-6F assay covers most common body fluids and cell types, and co-extracted DNA was detected in every sample [21]. There are several possible explanations for the high number of failed samples. First, the MPS method is not optimized for samples with low levels of RNA. Ideally, 25 ng of RNA should be reverse transcribed in the cDNA synthesis. Most of the samples in this study did not have such a high RNA content (Supplementary File S3). The RNA quantification method (Quantus^®^ Flurometer, Promega, Madison, WI, USA) used was not human-specific; hence, the actual quantity of human RNA input into the assay could be less than intended due to contaminating non-human RNA (bacterial RNA content). It is known that vaginal mucosa contains bacterial RNA [23,24,25,26]. Several samples were diluted prior to cDNA synthesis due to high RNA quantification values, and some of these may have resulted in negative results with MPS due to the dilution. Another reason could be that remnants from the DNAse inactivation reagent were present in some RNA extracts after DNAse treatment. This reagent, or the presence of other PCR inhibitors, may inhibit downstream enzymatic reactions, especially with the MPS protocol [22,27]. The samples had been stored at negative 80 degrees for approximately 1 year prior to the analysis, and it is possible that the quality of the samples may have suffered. However, the re-analysis of 10 samples for which MPS had given negative results provided positive results with the CE method, albeit with some diminution of the signals and the number of detectable markers in some of them.

The RNA extracts were treated with DNAse to eliminate any residual DNA. In addition, the BFID-cSNPs-6F assay was designed not to amplify any genomic DNA (gDNA) present in a sample. The primers in the assay are either RNA-specific (i.e., one primer spanning an exon–exon junction) or located within the exons, but with the corresponding DNA product being too large to be generated (>200 bp). The exception for the latter is the PRM1 semen-specific marker, which contains a short intron, resulting in a gDNA amplicon of 191 nts compared to the RNA/cDNA amplicon of 100 nts [20]. In seven samples, a gDNA PRM1 marker was detected, albeit in small amounts (<1%) compared to the overall coverage of the sample. The ability to detect the gDNA PRM1 marker, and to distinguish it from the RNA-specific PRM1 marker, provides information about the level of residual genomic DNA, if any, present in the RNA samples. The design of the assay, combined with the software tool (TMAP), provides an additional benefit in that there is no need to run RT-negative controls (cDNA samples without RT enzyme).

In this study, the DNA quantity, measured in adjusted RFU values aligned with POI per locus, was significantly higher in samples with a positive result for vaginal mucosa compared to the samples with a negative test result (as shown with the CE method). The association between the DNA quantity and the mRNA vaginal mucosa test result was more distinct with the CE method. However, the combination of DNA STR analysis and RNA sequencing with MPS demonstrates the ability to identify the DNA contributors and the body fluids present in the sample, and, most importantly, to associate the detected body fluid with a DNA contributor.

Future research directions would ideally be to optimize the assay for low-level RNA samples. Our sample set, which represents realistic casework samples, consisted of samples collected hours after intimate contact or after non-intimate contact, and contained only traces of transferred biological material. For our samples, it would have been valuable to detect skin markers, especially in cases with low quantities of DNA, to investigate whether the DNA contribution from the POI derived from skin cells or from vaginal mucosa cells persisting from the original contact. Furthermore, since the current quantification methods are not human-specific, it would be useful to develop a body fluid-specific human RNA quantification method. The library/template preparation method used for the RNA samples was fully automated using the Ion Chef^TM^. The automated method has not been optimized for low template RNA samples. It would be worthwhile to investigate, using the alternative manual library/template preparation method, whether it is possible to better optimize these multistep processes to achieve improved analytical sensitivity of low template RNA samples.

## 5. Conclusions

In this study, we tested the performance of the BFID-cSNP-6F assay with the Ion S5™ System on 158 samples from fingernail swabs, penile swabs and boxershorts collected at different time points after intimate contact and after non-intimate contact. The analysis showed that the assay was able to perform well on these types of samples, and that the additional information obtained with the cSNP genotypes could successfully associate body fluids with contributors in most cases. We observed a lower success rate for the detection of vaginal mucosa with the MPS method compared to the CE method.

## Figures and Tables

**Table 1:** The percentage of samples (exact numbers in brackets) in which the various body fluids present in both assays (CE and MPS) were “detected” according to the respective scoring rules. The last column shows the total number of samples analyzed in each category (*n*).

Location	Time Point	Vaginal Mucosa		Blood		Semen		Saliva		Menstrual Blood		*n*
		CE	MPS	CE	MPS	CE	MPS	CE	MPS	CE	MPS	
Fingernail swabs	0	100% (12)	42% (5)	8% (1)	0	25% (3)	0	8% (1)	0	0	0	12
	12	45% (5)	45% (5)	0	0	0	0	0	18% (2)	0	0	11
	18	20% (2)	0	0	0	0	0	0	0	0	0	10
	24	9% (1)	0	9% (1)	0	0	0	9% (1)	0	0	0	11
	36	20% (2)	10% (1)	0	0	0	0	0	0	0	0	10
	Non-int. contact	0	0	0	0	0	0	0	0	0	0	12
Penile swabs	0	100% (12)	75% (9)	25% (3)	0	33% (4)	33% (4)	25% (3)	17% (2)	0	0	12
	12	92% (11)	42% (5)	0	0	42% (5)	50% (6)	0	0	0	0	12
	18	64% (7)	27% (3)	0	0	36% (4)	45% (5)	0	0	0	0	11
	24	27% (3)	9% (1)	0	0	36% (4)	27% (3)	0	0	0	0	11
	36	8% (1)	9% (1)	0	0	27% (3)	27% (3)	0	0	0	0	11
	Non-int. contact	0	0	0	0	8% (1)	17% (2)	0	0	0	0	12
Boxer- shorts	0	82% (9)	55% (6)	18% (2)	0	64% (7)	27% (3)	0	0	0	0	11
	Non-int. contact	8% (1)	0	0	0	17% (2)	8% (1)	0	0	0	0	12
Total		68	36	7	0	33	27	5	4	0	0	158

**Table 2:** The percentage of samples (exact numbers in brackets) positive for vaginal mucosa, semen, saliva and skin for which the cSNP genotypes could associate the body fluid with the person of interest (POI—female partner), the donor (male partner), or called “Inconclusive” (Incon.). The last row shows the total number of samples in each category for each body fluid.

Location	Time Point	Vaginal Mucosa			Semen			Saliva			Skin		
		POI	Donor	Incon.	POI	Donor	Incon.	POI	Donor	Incon.	POI	Donor	Incon.
Fingernail swabs	0	100% (5)	0	0	0	0	0	0	0	0	0	0	0
	12	80% (4)	0	20% (1)	0	0	0	0	0	100% (2)	0	0	100% (1)
	18	0	0	0	0	0	0	0	0	0	0	0	0
	24	0	0	0	0	0	0	0	0	0	0	0	0
	36	100% (1)	0	0	0	0	0	0	0	0	0	0	0
	Non-int. contact	0	0	0	0	0	0	0	0	0	0	0	0
Penile swabs	0	100% (9)	0	0	0	100% (4)	0	0	50% (1)	50% (1)	0	0	0
	12	100% (5)	0	0	0	83% (5)	17% (1)	0	0	0	50% (1)	50% (1)	0
	18	100% (3)	0	0	0	80% (4)	20% (1)	0	0	0	0	33% (1)	67% (2)
	24	100% (1)	0	0	0	67% (2)	33% (1)	0	0	0	0	0	0
	36	100% (1)	0	0	0	67% (2)	33% (1)	0	0	0	0	100% (3)	0
	Non-int. contact	0	0	0	0	100% (2)	0	0	0	0	0	100% (1)	0
Boxershorts	0	100% (6)	0	0	0	100% (3)	0	0	0	0	0	0	0
	Non-int. contact	0	0	0	0	100% (1)	0	0	0	0	0	100% (1)	0
Total		97% (35)	0	3% (1)	0	85% (23)	15% (4)	0	25% (1)	75% (3)	9% (1)	64% (7)	27% (3)

**Table 3:** cSNP genotypes observed in penile swabs collected 24 h and 0 h post-intimate contact from two different couples (**RNA-cSNP**), the number of reads and the genotypes of the reference sample (**DNA-cSNP**). Donor refers to the male partner and POI to the female partner. mRNA markers with no reads are not shown. The cells in grey show the genotypes that only corresponded to one of the two reference samples.

Sample	Marker	cSNP	RNA-cSNP		DNA-cSNP	
			Alleles	Coverage	Donor	POI
Penile swab collected 24 h post-intimate contact	Semen_KLK3	rs11573	**TT**	1125	**TT**	**TT**
	Semen_KLK3	rs1135766	**AA**	1125	**AA**	**AA**
	Semen_SEMG2	rs2233896	**AC**	177	**AC**	**CC**
	Semen_TGM4	rs1995640	**CC**	906	**CC**	**CT**
	Semen_TGM4	rs1995641	**GG**	2586	**GG**	**AG**
	Semen_TGM4	rs3749195	**CT**	393	**CT**	**CT**
	Semen_TGM4	rs9876921	**AG**	393	**AG**	**AG**
	Vaginal mucosa_CYP2B7P1	-	**-**	178	**-**	**-**
	Vaginal mucosa_MUC22	rs3869098	**AG**	755	**GG**	**AG**
	Vaginal mucosa_MUC22	rs4248153	**AG**	755	**GG**	**AG**
	Vaginal mucosa_MUC22	rs1419664	**CT**	542	**CC**	**CT**
	Vaginal mucosa_MUC22	rs3094672	**AA**	379	**AA**	**AA**
	Vaginal mucosa_MUC22	rs12110470	**GG**	88	**GG**	**GG**
	Vaginal mucosa_MUC22	rs12110785	**TT**	88	**TT**	**TT**
	Vaginal mucosa_MUC22	rs10947121	**CT**	165	**CT**	**CT**
Penile swab collected 0 h post-intimate contact	Saliva_MUC7	rs2306948	**CC**	455	**CC**	**CC**
	Saliva_STATH	-	**-**	357	**-**	**-**
	Vaginal mucosa_CYP2A6	rs8192721	**CC**	603	**CT**	**CC**
	Vaginal mucosa_CYP2B7P1	-	**-**	26,353	**-**	**-**
	Vaginal mucosa_MUC22	rs3869098	**GG**	12,559	**AA**	**GG**
	Vaginal mucosa_MUC22	rs4248153	**AG**	15,550	**AA**	**AG**
	Vaginal mucosa_MUC22	rs1419664	**CC**	12,367	**CC**	**CC**
	Vaginal mucosa_MUC22	rs3094672	**AA**	12,267	**TT**	**AA**
	Vaginal mucosa_MUC22	rs12110470	**GG**	2411	**GG**	**GG**
	Vaginal mucosa_MUC22	rs12110785	**TT**	2411	**TT**	**TT**
	Vaginal mucosa_MUC22	rs10947121	**CT**	2669	**TT**	**CT**

## Data Availability

The data supporting the findings reported in this manuscript can be found in Supplementary Files S2 and S3.

## Supplementary Materials

The following files are available online at: https://www.mdpi.com/article/10.3390/genes14030636/s1, Supplementary File S1, Table S1: Information of mRNA markers and cSNPs included in the panel; Table S2: The detection of gDNA PRM1 marker in seven samples; Figure S1: Dot plot showing the number of reads of the different body fluids detected in each sample; Figure S2: Scatter plot displaying the association of total reads per profile including all body fluid markers and RNA quant in samples; Figure S3: Scatter plot displaying the association of the number of reads for vaginal mucosa markers and RNA quantity in samples; Figure S4: The number of reads at each target for CYP2A6, CYP2B7P1 and MUC22 split into different time points and location of sampling; Figure S5: Scatter plot displaying the number of reads for the different vaginal mucosa cSNPs; Figure S6: Distribution of heterozygous balance observed in the cSNP genotypes for the different mRNA markers. Supplementary File S2: Data of cSNPs. Supplementary File S3: Data of MPS and CE methods.
