# Supplementary material for: Body Fluid Identification in Samples Collected after Intimate and Social Contact: A Comparison of Two mRNA Profiling Methods and the Additional Information Gained by cSNP Genotypes
Source: Genes (Basel). 2023 Mar 3;14(3):636. doi: 10.3390/genes14030636 (PMC10048544; doi:10.3390/genes14030636)
Supplement: Supplementary file 1 [file genes-14-00636-s001.zip › Supplementary File S1.pdf]

# Supplementary File S1 to « Body fluid identification in samples collected after intimate and social contact: a comparison of two mRNA profiling methods and the additional information gained by cSNP genotypes»

**Supplementary Table S1**: Information of mRNA markers and cSNPs included in the panel

| Body fluid | mRNA marker | Chr | cSNP | Location (GRCh38) | Alleles[1] | MAF[2] |
|---|---|---|---|---|---|---|
| Blood | ANK1 | 8 | rs504574 | 41696410 | C>A,G | 0.4071 (G) |
| | ANK1 | 8 | rs7816734 | 41690230 | G>A | 0.1873 (A) |
| | CD3G | 11 | rs3753059 | 118350634 | T>C | 0.2963 (C) |
| | SPTB | 14 | rs1741487 | 64779244 | A>G | 0.4119 (G) |
| | SPTB | 14 | rs1741488 | 64779238 | C>A,T | 0.4115 (T) |
| | SPTB | 14 | rs229586 | 64796629 | C>G,T | 0.2813 (T) |
| | SPTB | 14 | rs229592 | 64774510 | A>C,G,T | 0.4784 (G) |
| Menstrual blood | LEFTY | 1 | 0 | - | - | - |
| | MMP10 | 11 | rs17860949 | 102779515 | G>A | 0.1194 (A) |
| | MMP10 | 11 | rs17860950 | 102779223 | A>G | 0.0751 (G) |
| | COL12A1 | 6 | rs240736 | 75138465 | A>G | 0.2650 (G) |
| | COL12A1 | 6 | rs594012 | 75132006 | A>C,T | 0.1138 (A)* |
| | COL12A1 | 6 | rs970547 | 75087586 | C>A,G,T | 0.2881 (C)* |
| | COL6A3 | 2 | rs1131296 | 237334649 | G>A | 0.3462 (A) |
| | COL6A3 | 2 | rs2270669 | 237334821 | C>A,G,T | 0.2077 (C)* |
| | COL6A3 | 2 | rs4433949 | 237340987 | C>A,T | 0.3425 (T) |
| | COL6A3 | 2 | rs34558385 | 237341074 | G>A,C,T | 0.0435 (A) |
| | COL6A3 | 2 | rs3790993 | 237350171 | C>G,T | 0.4457 (C)* |
| | MMP3 | 11 | rs679620 | 102842889 | T>A,C,G | 0.3478 (T)* |
| Saliva | HTN3 | 4 | rs1849937 | 70033190 | C>T | 0.1124 (T) |
| | HTN3 | 4 | rs1136515 | 70033236 | C>T | 0.4798 (C)* |
| | HTN3 | 4 | rs75067954 | 70036304 | C>T | 0.0234 (T) |
| | MUC7 | 4 | rs2306948 | 70474011 | C>T | 0.2087 (T) |
| | PRB4 | 12 | rs1052808 | 11308358 | C>G,T | 0.2450 (C)* |
| | PRH2 | 12 | rs10772391 | 10931078 | T>A,C | 0.2175 (T)* |
| | STATH | 4 | 0 | - | - | - |
| Semen | KLK3 | 19 | rs11573 | 50856241 | T>A,C | 0.3399 (C) |
| | KLK3 | 19 | rs1135766 | 50856247 | A>C,G | 0.3399 (G) |
| | PRM1 | 16 | rs737008 | 11281009 | G>A,T | 0.4878 (G)* |
| | SEMG2 | 20 | rs2233896 | 45221759 | C>A,G | 0.0992 (A) |
| | TGM4 | 3 | rs1995640 | 44901577 | C>A,T | 0.4327 (C)* |
| | TGM4 | 3 | rs1995641 | 44901897 | G>A,T | 0.4449 (G)* |
| | TGM4 | 3 | rs3749195 | 44906987 | C>T | 0.3255 (C)* |
| | TGM4 | 3 | rs9876921 | 44907098 | G>A,C | 0.3806 (G)* |
| Skin | COL17A1 | 10 | rs805701 | 104060198 | G>A | 0.4475 (G)* |
| | IL37 | 2 | rs3811046 | 112913801 | G>A,C,T | 0.4000 (G)* |
| | IL37 | 2 | rs3811047 | 112913833 | A>G,T | 0.3826 (A)* |
| | LCE1C | 1 | rs36107483 | 152805392 | A>C,G | 0.1869 (G) |
| | LCE1C | 1 | rs2006940 | 152805432 | C>A,T | 0.2206 (T) |
| | LCE1C | 1 | rs17624493 | 152805488 | G>A | 0.1597 (A) |
| Vaginal mucosa | CYP2A6 | 19 | rs8192721 | 40850283 | C>T | 0.0611 (T) |
| | CYP2B7P1 | 19 | 0 | - | - | - |
| | MUC22 | 6 | rs3869098 | 31034675 | A>C,G,T | 0.4315 (A)* |
| | MUC22 | 6 | rs4248153 | 31034750 | A>C,G,T | 0.4982 (G) |
| | MUC22 | 6 | rs1419664 | 31025536 | C>T | 0.1206 (T) |
| | MUC22 | 6 | rs3094672 | 31025600 | T>A,C,G | 0.2040 (T)* |
| | MUC22 | 6 | rs12110470 | 31030039 | G>C,T | 0.2829 (T) |
| | MUC22 | 6 | rs12110785 | 31030047 | T>C | 0.1579 (C) |
| | MUC22 | 6 | rs10947121 | 31032220 | T>A,C,G | 0.4531 (C) |

1 Alleles in designated SNP
2 Minor Allele Frequency (or frequency*) reported in the 1000 Genomes project.

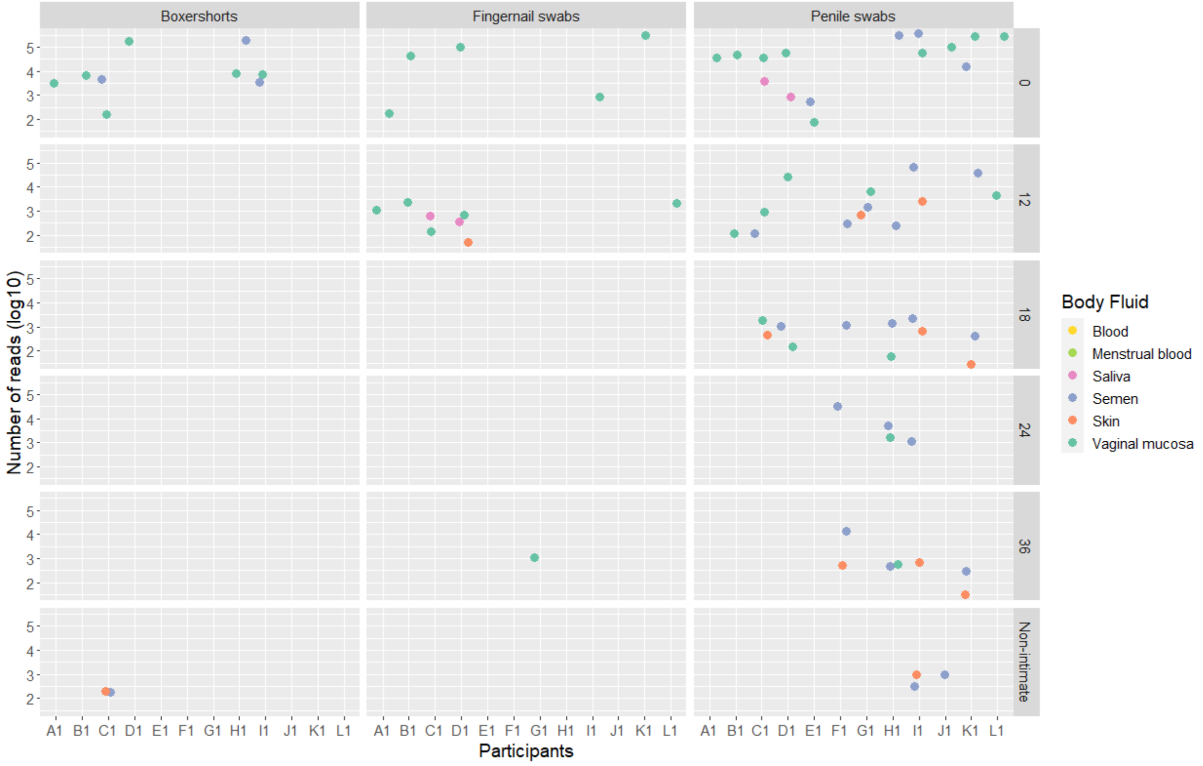


**Figure S1**: Dot plot showing the number of reads (log 10) of the different body fluids detected in each sample, divided into participants, sample location and time of sampling, N=52. There were no positive results for blood or menstrual blood.

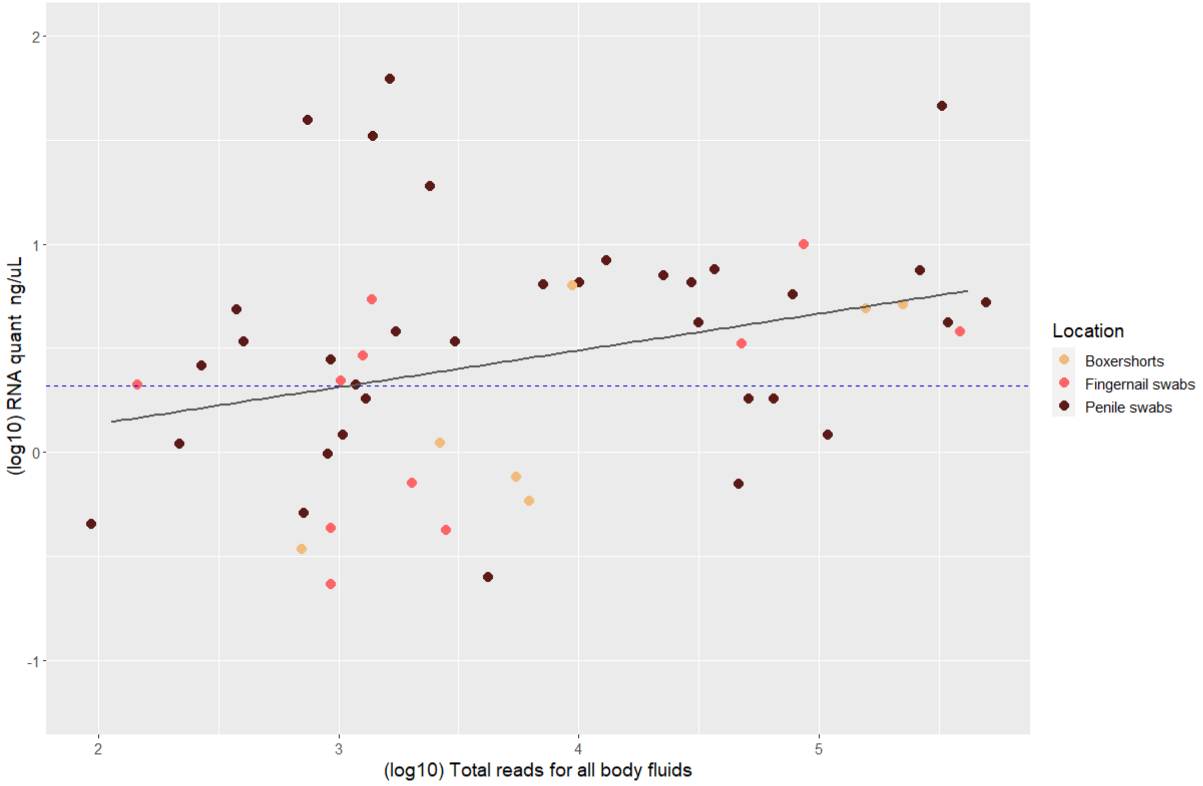


**Figure S2**: Scatter plot displaying the association of total reads (log10) per profile including all body fluid markers and RNA quant (log 10) in samples. The dark grey line represent the regression line, while the dashed line (blue) shows the threshold of reaching the optimized amount of RNA (25 ng) to the RT reaction, N=52.

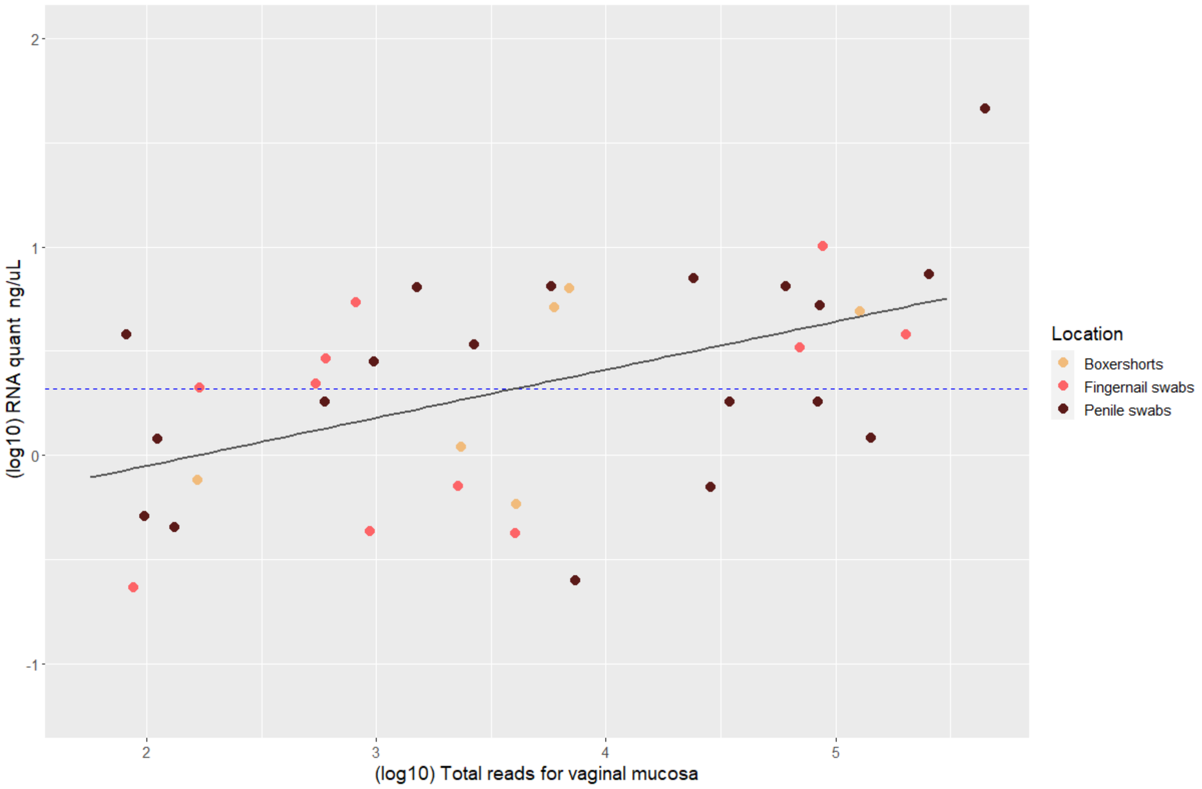


**Figure S3**: Scatter plot displaying the association of the number of reads (log10) for vaginal mucosa markers and RNA quantity (log 10) in samples. The dark grey line represent the regression line, while the dashed line (blue) shows the threshold of reaching the optimized amount of RNA (25 ng) to the RT reaction, N=36.

**Supplementary Table S2**: The detection of gDNA PRM1 marker in seven samples

| Participant | Sample | Time point | gDNAPRM 1 reads | rnaPRM1 reads | RNA Total reads | % gDNA of rnaPRM1 marker | % gDNA of total reads |
|---|---|---|---|---|---|---|---|
| F1 | Penile swab | 24 | 306 | 29980 | 31576 | 1,02 | 0,97 |
| F1 | Penile swab | 36 | 87 | 9469 | 13938 | 0,92 | 0,62 |
| I1 | Penile swab | 0 | 203 | 97542 | 419848 | 0,21 | 0,05 |
| I1 | Penile swab | 12 | 362 | 57431 | 69976 | 0,63 | 0,52 |
| I1 | Boxershorts | 0 | 5 | 556 | 10809 | 0,9 | 0,05 |
| K1 | Fingernail swab | 0 | 31 | 4260 | 310073 | 0,73 | 0,01 |
| K1 | Penile swab | 12 | 28 | 19621 | 37711 | 0,14 | 0,07 |

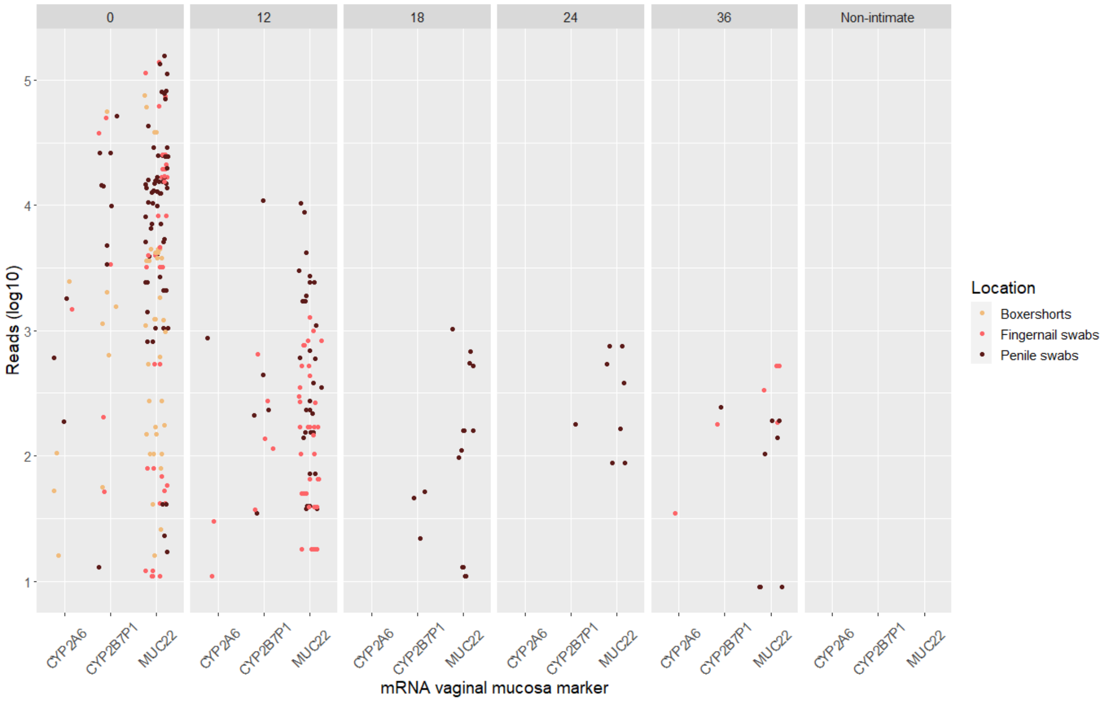


**Figure S4**: The number of reads (log10) at each target for CYP2A6 (1 cSNP), CYP2B7P1 (1 target) and MUC22 (7 cSNPs) split into different time points and location of sampling.

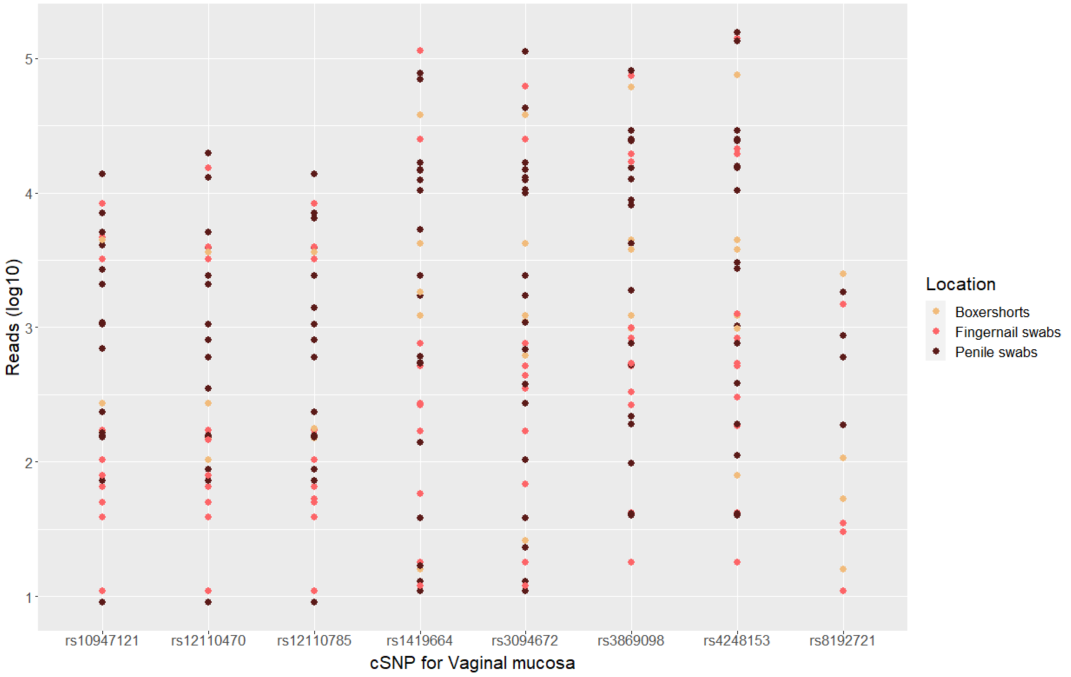


**Figure S5**: Scatter plot displaying the number of reads (log10) for the different vaginal mucosa cSNPs. rs8192721 (far right) belong to mRNA marker CYP2A6, while the others belong to MUC22, N=36.

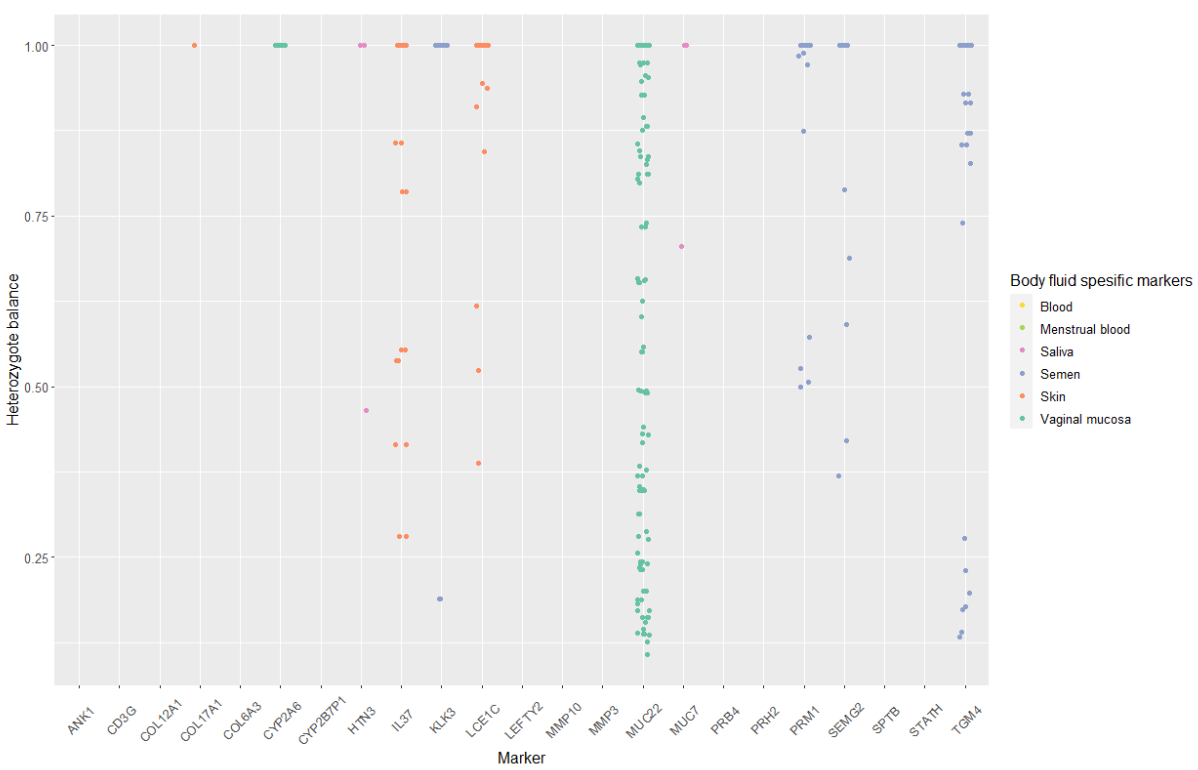


**Figure S6**: Distribution of heterozygous balance observed in the cSNP genotypes for the different mRNA markers.
